# The structure and functional correlates of social support networks of people in advanced old age living in chosen urban and rural areas in Poland: a cross-sectional study

**DOI:** 10.1007/s10433-020-00583-6

**Published:** 2020-10-16

**Authors:** Z. B. Wojszel, B. Politynska

**Affiliations:** 1 Department of Geriatrics, Medical University of Bialystok, Fabryczna str. 27, 15-471 Bialystok, Poland; 2 Department of Geriatrics, Hospital of the Ministry of Interior in Bialystok, Bialystok, Poland; 3 Department of Philosophy and Human Psychology, Medical University of Bialystok, Szpitalna str. 37, Bialystok, Poland

**Keywords:** Social support network, Health correlates, Functional disability, Physical and mental abilities, Practitioner assessment of network type

## Abstract

The purpose of the study was to identify the different types of social support networks (SSNs) among community-dwelling people aged 75+ years in selected areas of Poland, and to evaluate any associations between the network type and demographic and health variables of the population studied. The two most prevalent SSN types identified using the Practitioner Assessment of Network Type were “family dependent” (35.8%) and “locally integrated” (32.2%). “Local self-contained” (6.4%), “wider community focused” (2.8%) and “private restricted” (5.6%) SSNs were observed less frequently. In 17.2% of cases, it was not possible to identify the type of network unequivocally. Older people with a locally integrated SSN, in contrast to the family dependent type, were generally younger, living alone, and less likely to be homebound, rate their health as poor, suffer from depression or dementia, and had lower levels of functional disability. Locally integrated SSNs are recognized in the literature as being the most robust in terms of facilitating well-being and providing sufficient support to help maintain the older person in the community. This may reflect the higher levels of independence of older people able to sustain these support networks, which are then transformed into family-dependent types as their health deteriorates, but confirmation of this would require prospective studies. An improved understanding of the prevalence of different types of social networks among older people in Poland would help to guide a systematic approach to recognizing unmet needs in this population and provide crucial information in the planning of formal services.

## Introduction

Gerontologists have long been interested in the role of social networks in providing support for older adults, with a view to gaining a better understanding of the impact on their health and well-being (Berkman et al. 1992; Bosworth and Schaie 1997). Assessment of social networks and social support is recognized as an important component of a geriatric assessment protocol, since having a network that is able to provide instrumental and emotional social support, can affect the decisions made by the geriatric team caring for an older person with regard to future care and placement (Kane 1995).

*Social* networks are formed by people with whom individuals are in some type of relationship, and determine their participation in social groups and society more generally, while *support* networks refer to the network participants and their ability to provide help, support and advice. Older people’s capacity to cope with life and its problems is related to the structure and content of their social support networks (SSNs) (Wenger and Tucker 2002), whose strength is recognized by policy-makers as important for predicting community care outcomes (Faber Ashley and Wasserman 2002). In view of the projected increase in the number and proportion of older people in Poland (Central Statistical Office 2014), this has important implications for both the family and society as whole, particularly with respect to the capacity for providing long-term care.

There are various models of informal support networks, and specific instruments derived from the resultant typologies have been applied to older populations in order to assess the various effects of network type (Burholt and Dobbs 2014; Litwin 1997; Lubben and Gironda 2004; Park et al. 2015). The instruments share common features, such as frequency of contact with or proximity to children, but differ according to focus, some measuring social isolation and levels of perceived social support from family and friends, others attempting to categorize these relationships and relating specific categories to outcomes in terms of health, functioning and psychological well-being.

The majority of studies on the SSNs of older people have been carried out in Western Europe, little work having been reported to date in East European countries, in Poland in particular. Some relevant findings concerning older people’s social integration and the extent of home care provision were provided by the Polish National Gerontological Study (Synak 2002) and the PolSenior study (Bledowski et al. 2011), which examined sources of support for older people, but neither study attempted to create a typology of support networks. An important finding from these studies was that the social potential of older people and availability of care resources differed significantly between rural and urban settings.

The Collaborative Research on Ageing in Europe Project (COURAGE), completed in 2011 in three European countries: Poland, Spain and Finland, was one of the first to evaluate SSNs in people aged 50+ in the Polish context. The COURAGE Social Network Index assessed the construct of social networks based on an evaluation of their functioning (frequency of direct contact, ties and social support) (Leonardi et al. 2014) and proved useful in identifying high risk groups with weaker social networks (Zawisza et al. 2014). Nevertheless, a typology of SSNs was not provided and only a summative assessment was made of their quality. This cumulative quality indicator was lower among women, those living in urban areas and decreased with age (Tobiasz-Adamczyk and Zawisza 2017). No information, however, was provided on specific risk factors associated with the result.

The Practitioner Assessment of Network Type (PANT) (Wenger 1994a) addresses these questions by identifying the support needs of older people and determining those at greatest risk of a breakdown in care, thus helping to inform the planning of community and social care services (Wenger 1997b; Wenger and Tucker 2002). It assesses the different dimensions of networks and produces a typology of five network types that differ in their ability to provide support for community dwelling older people. The two most common SNNs are the family-dependent and locally integrated types, which account for more than half of those identified with the PANT instrument (Drennan et al. 2008; Golden et al. 2009). The family-dependent network is relatively small, based on close, proximate family ties (usually with a daughter), but little outside involvement. The dependency of the older person is higher than in other types of SSNs (Wenger 1994b), being associated with advanced old age, dementia, poorer health status and greater mortality risk (Santini et al. 2015); social involvement is low, and there is greater risk of loneliness, depression and other mental health difficulties. Locally integrated SSNs are somewhat larger with greater structural variability, based on close relations with nearby family, friends and neighbours built up over many years of active participation in community life (Wenger and Tucker 2002). Long-standing mutually beneficial interactions allow the person to seek assistance from the network insofar as they remain independent, which affords protection against adverse mental health outcomes and isolation (Wenger 1994a).

The three remaining types of SSNs, where family members do not live in close proximity, have a lower ability to deal with disability and dependency in old age. The local self-contained types are associated with infrequent family contact and greater reliance on neighbours, especially in emergencies, though expectations of support are few and help is resisted unless it is unavoidable, when recourse to statutory services is preferred. Inevitably, both physical and mental health problems are recognized late and are often crisis related (Wenger 1997a,b). Wider community-focused networks are relatively large, based mainly on the involvement of friends and voluntary groups which provide high levels of emotional support, so that the older person does not suffer from depression or social isolation and remains largely independent and self-sufficient. Problems arise when increased levels of frailty and loss of mobility lead to diminished social participation which frequently herald loneliness and its contingent difficulties. Private-restricted networks are small, characterized by low levels of participation from family and friends and few community contacts. Such networks are more common for people in advanced old age, often widowed, who have outlived friends and other social contacts and whose families do not live locally and those with a history of mental illness. Social isolation is often extreme leading to increased rates of low morale, loneliness and depression in comparison with other types of networks (Drennan et al. 2008).

The support network typology proposed by Wenger has high predictive validity, individual network types being associated with specific demographic and outcome variables (Wenger 1997a) predictive of: mental health outcomes (Drennan et al. 2008), health, loneliness and isolation (Stephens et al. 2011), morale (Wenger and Shahtahmasebi 1991), hospital discharge outcomes, benefit take-up, residential care placement (Wenger 1997b), poorer health and isolation (Thiyagarajan et al. 2014) and mortality (Santini et al. 2015). The PANT’s validity has been confirmed in different populations (Stephenson et al. 2011; Szabo et al. 2018; Thiyagarajan et al. 2014; Wenger and Tucker 2002), and the instrument is available with a training package and is user-friendly, a major advantage being that it can be easily implemented by practitioners in routine clinical practice. PANT allows approximately 75% of networks to be classified unequivocally, with a further 20% described as “borderline”, showing the characteristics of two types (Wenger 1997a). The remaining 5% may be unclassifiable as frequently occurs for people with cognitive impairment, which itself is a high risk factor, contributing to detrimental outcomes and limiting capacity for independent living.

## Purpose

The purpose of this study was to examine the frequency of different types of SSNs as defined by the PANT and to evaluate the association with selected demographic and health variables in people of advanced old age (75+ years) living in chosen areas of Poland. In keeping with the research findings outlined above, it was hypothesized that network type would be associated with the older person’s health outcomes including subjective well-being, cognitive ability and functional disability level. To the best of the authors’ knowledge, this is the first study concerning the SSNs of people in advanced old age to be carried out in Poland using the PANT instrument.

## Methods

The study formed part of a multidimensional research project performed in 2009 based on a cross-sectional questionnaire into geriatric disability syndromes and availability of supporting services in advanced old age, the details of which have been reported elsewhere (Wojszel 2009). The population studied lived in two selected areas (urban—Bialystok city, and rural—Sokolka community) of the Podlaskie region in north-eastern Poland, a borderland area with a relatively multi-ethnic structure, which is one of the demographically oldest Polish provinces; persons aged 65 years or more constituted 14.7% and those over 75 years, 7.1% of the population (Central Statistical Office 2008). It is an economically deprived region, characterized by low levels of urbanization, which in 2005 had the lowest gross domestic product per capita in the European Union (Central Statistical Office 2011) and consequently received special additional support from European Funds (Operational Programme 'Development of Eastern Poland' 2007–2013).

Bialystok is the largest urban agglomeration in north-eastern Poland with about 300,000 inhabitants. It is the administrative, economic, scientific and cultural centre of the region. Sokolka is one of ten typical agricultural municipalities of the surrounding region, where 28.4% of the population live in villages, and is the seat of the municipal authorities.

The present study was planned as a replication of a cross-sectional study carried out in 2000 in the same areas and based on a random sample identified from the database of national records for the inhabitants of this region—the Universal Electronic System for Registration of the Population (PESEL) (Bień et al. 2001). However, in common with other researchers (Wyka et al. 2012), an attempt to select eligible persons (75+ years) in this way yielded an unacceptably low response rate, likely to undermine the representativeness of the sample. Our study was thus carried out on a sample matched precisely in terms of age and gender to the demographic structure of the population living in the studied areas, on the basis of data from the Central Statistical Office (2008). The sample size for Bialystok was determined for individual districts (separately) in proportion to the population and in accordance with age and gender distributions. In Sokolka a quota sample was obtained among residents scattered throughout villages in the rural community, provided that the selected respondents met the conditions for participation in the study in relation to age and gender distribution. A total of 509 community dwelling older people aged 75 years or more (253 from rural and 256 from urban areas) took part in the study. Face-to-face interviews were carried out in respondent’s homes by trained interviewers from the Research Institute of the Polish Sociological Association. Where the eligible respondent could not be interviewed, often because of medical or cognitive problems, a proxy—the caregiver—was enlisted to answer questions on their behalf. This occurred for 63 people (12.4% of the entire sample), 26 (10.2%) of whom lived in towns and 37 (14.6%) in villages. Of these, 69.8% were aged 80+ years, 80.4% had dementia, and all were classified disabled in terms of activities of daily living (ADL).

The assessment included questions concerning: the older person’s socio-demographic characteristics i.e. age, gender, marital status, residence (urban/rural), living arrangements (alone/with others), education level, self-rated economic status; subjective health status (self-rated on a 5-point Likert scale from poor to excellent), information obtained from the respondent or caregiver regarding 16 listed chronic illnesses (including ischemic heart disease/myocardial infarction, chronic cardiac failure, hypertension, stroke, chronic pulmonary disease, diabetes, peptic ulcers, liver disease/cholelithiasis, neoplasms, parkinsonism, chronic arthritis, diseases affecting the kidneys, eyes (e.g. glaucoma, cataracts), thyroid gland, depression and injuries/fractures), routine medication over previous 3 months and mobility. Geriatric functional ability scales and an evaluation of the respondent’s SSN were also included. Economic status was self-evaluated as either good, satisfactory, poor or unable to say, and level of education classified as: complete/incomplete primary education, vocational/incomplete secondary education, complete secondary/with school leaving examination, post-secondary education, higher education—Bachelor’s/Master’s degree.

The respondent’s functional ability was assessed using:

The EASY-Care system (Philp 1997), constructed on the basis of ten ADL from the Barthel scale (Mahoney and Barthel 1965) and the six-item instrumental IADL scale from the Duke OARS assessment (Fillenbaum and Smyer 1981), plus an item on mobility. A “functional disability index” (FDI) was calculated on the basis of the EASY-Care items, which were recoded (1 = unable or only able with help/incontinent versus 0 = able without help/continent), as appropriate. The score on the index ranged from 0 to 17 (greatest disability).

The mobility scale (Piotrowski 1973), classifying respondents as: I—able to walk in and outside the home; II—able to walk around the home, but having difficulties outside; III—able to walk around the home, but unable to go outside; IV—bedridden, in a wheelchair, or confined to an armchair. Those in categories III and IV were classified as “homebound”.

Cognitive status was assessed with the Short Orientation-Memory-Concentration test (Katzman et al. 1983). Point scores were dichotomized, 0–10 considered normal to mild, and above 10 (maximum 28 points), as moderate or serious cognitive impairment. The complete test was performed with 196 (76.6%) urban and 204 (80.6%) rural respondents. An estimate of cognitive ability was also possible in 3 cases of diagnosed dementia, treated with pro-cognitive drugs, which were included in the analyses using the corrected variable “cognitive impairment”. Forty-eight respondents (9.4%) refused the test, and in 44 cases (8.6%), it was not performed because of the respondent’s poor health status.

The Geriatric Depression Scale (GDS) (Yesavage 1988) was used to identify depression in the respondents. A dichotomous classification with 0–5 points considered normal and 6–15 points indicating possible depression was adopted. In 23 cases, despite incomplete test results, the affective status of the respondent was determined on the basis of other information; this included two respondents with a medical diagnosis of depression and one taking prescribed antidepressant medication. The corrected variable “depression” was used in analyses.

The PANT was used to identify the SSNs of older people (Wenger 1994a). It allows the type of SSN to be identified on the basis of responses to eight pre-coded questions which explore: distance to nearest relative (excluding spouse), child or sibling and contact frequency with children or other relatives, friends in the community/ neighbourhood, neighbours, religious involvement and involvement in community or social groups. The tool was translated into Polish, back-translated into English, and then culturally validated before its use in the research. We did not conduct cluster analyses to develop network types, but followed the rules of response categories coding and instructions for the interpretation of the PANT results available in the training package (Wenger 1994a). Replies to every question in the PANT are coded, allowing them to be assigned to different network types. The codes which dominate the pattern of responses determine the network type for the respondent, or the type of borderline case observed.

### Statistical analysis

Data analyses were conducted using the IBM SPSS Version 18 Software suit (SPSS, Chicago, IL, USA). Median values and interquartile ranges for non-normally distributed continuous variables and the number of cases and percentages for categorical variables were calculated. Variable distributions were tested using the Kolmogorow–Smirnow test. Regression analyses were performed to assess the relationship between the network types (specified as predictors) and their health correlates. Six categories of the variable “support network” were recoded as dummy variables, and 5 of its 6 categories were included in the regression models. This allowed us to evaluate the correlates of the various SSNs in comparison with the reference category of “family dependent”, as the most frequently observed network type. A p value of ≤ 0.05 was regarded as significant.

## Results

The demographic and health-related characteristics of the respondents are summarized in Table 1. The majority of respondents were women, over 80 years old, living with others, and with primary or lower levels of education. Although a high percentage of respondents rated their health status as poor, reported visual and hearing impairments, were classified as cognitively impaired or depressed, the median score of their FDI was in the moderate range. Homebound people constituted 20% of the study group.

**Table 1:** Characteristics of the study group

Characteristic	Frequencies (%) or medians (IQR)	Missing data
	No. = 509	
Age, years	80 (77–83)	–
80+ year old	266 (52.3)	–
Gender, female	346 (68.0)	–
	253 (49.7)	–
Marital status, married/cohabiting	150 (29.6)	3
Living alone	168 (33.2)	3
Education, secondary or above	120 (24.0)	10
Economic status, poor	118 (28.5)	95
Subjective health status, poor	200 (39.4)	2
Functional disability index (ADL) [0–17]	3 (0–7)	37
Cognitive impairment	182 (39.7)	49
Katzman’s scale score [0–28]	8 (4–14)	109
Depression	225 (45.8)	18
GDS score [0–15]	5 (2–10)	36
Mobility impairment, homebound	101 (19.8)	–
Number of drugs taken every day [0–15]	4 (2–6)	–
Number of chronic conditions [0–16]	3 (2–4)	8

*IQR* interquartile range; *GDS* Geriatric Depression Scale; *ADL* Activities of Daily Living

Table 2 shows how the samples responded to each of the PANT statements. The majority of older people reported having a family member living in close proximity, usually a child. This influenced the frequency of seeing the child or other relative i.e., primarily on a daily basis. The frequency of contact with family members was significantly greater than with friends or neighbours. 15.7% of participants reported either absence or lack of contact with friends and 7.8% had no contact with neighbours. The majority of respondents participated—at least occasionally—in religious meetings.

**Table 2:** Defining characteristics of the network

Characteristic	Frequency (%)
Nearest child or other relative	508 (100)
Same house/ within 1 mile	331 (65.2)
1–15 miles	129 (25.4)
More than 15 miles	40 (7.8)
No relatives	8 (1.6)
Nearest child	507 (100)
Same house/ within 1 mile	280 (55.2)
1–15 miles	130 (25.6)
More than 15 miles	44 (8.7)
No children	53 (10.5)
Nearest sibling	505 (100)
Same house/ within 1 mile	60 (11.9)
1–15 miles	142 (28.1)
More than 15 miles	122 (24.2)
No sisters or brothers	181 (35.8)
Frequency of seeing child/ relative	499 (100)
Daily	306 (61.3)
At least weekly	110 (22.0)
Less often	70 (14.0)
Never/ no relative	13 (2.6)
Frequency of seeing/ chatting with a friend	503 (100)
Daily	90 (17.9)
At least weekly	210 (41.7)
Less often	124 (24.7)
Never/ no friends	79 (15.7)
Frequency of seeing/ chatting with a neighbour	500 (100)
Daily	133 (26.6)
At least weekly	234 (46.8)
Less often	94 (18.8)
No contact with neighbours	39 (7.8)
Attends religious meetings	504 (100)
Yes, regularly (at least one a month)	222 (44.0)
Yes, occasionally	160 (31.7)
No	122 (24.2)
Attends community groups	489 (100)
Yes, regularly (at least one a month)	16 (3.3)
Yes, occasionally	32 (6.5)
No	441 (90.2)

All PANT questions were answered by 466 respondents (91.6% of the entire group studied). A support network could be identified unequivocally for 82.5% (Table 3). “Family dependent” and “locally integrated” networks constituted 73.8% of SSNs in rural areas and 62.4% in urban areas (*p* = 0.008). A high percentage of unclassified SSNs were borderline between two network types, the most frequent being “locally integrated—family dependent” or “local self-contained and private—family dependent”. In 1.7% of cases, the networks could not be classified because of contradictory data (inconclusive cases).

**Table 3:** Social support network types in the study group

Type of SSN	Frequencies (%)
	No. = 466
**Classified**	**386 (82.8)**
Family dependent (FD)	167 (35.8)
Locally integrated (LI)	150 (32.2)
Local self-contained (LS-C)	30 (6.4)
Wider community focused (WCF)	13 (2.8)
Private restricted (PR)	26 (5.6)
**Unclassified (U)**	**80 (17.2)**
Family dependent–locally integrated	34 (7.3)
Family dependent–private-restricted	11 (2.4)
Family dependent–local self-contained	6 (1.3%)
Locally integrated–local self-contained	9 (1.9)
Locally integrated–wider community focused	5 (1.1)
Locally integrated–private restricted	1 (0.2)
Local self-contained–wider community focused	3 (0.6)
Local self-contained–private restricted	1 (0.2)
Wider community focused–private restricted	2 (0.4)
Inconclusive cases	8 (1.7)

*SSN* social support network

The regression analyses (Table 4) confirmed that having a locally integrated SSN was connected with a greater likelihood of living alone and being better educated, with significantly lower odds for being older, having depression or dementia, rating one’s health as poor, being homebound, and having a lower score on the FDI. Equally, those with locally self-contained and wider community-focused SSNs were more likely to be living alone, were better educated, less likely to be depressed and had lower scores on the FDI. Only those with a locally self-contained SSN were less likely to be homebound in comparison with the reference group. People with private-restricted SSNs were also more likely to be living alone, but not in rural areas. Those with an unclassified SSN were more likely to be living alone and were better educated, but less likely to reside in a rural area, have depression or dementia, and were unlikely to have lower scores on the FDI.

**Table 4:** Logistic regressions on demographic and health correlates of social support networks*

		FD	LI	LS-C	WCF	PR	U
Age (80+ years)	OR (CI)	Ref.	0.58 (0.37–0.91)	0.81 (0.37–1.76)	0.82 (0.27–2.55)	0.82 (0.36–1.88)	0.61 (0.35–1.04)
	*p*		**0.02**	0.59	0.73	0.64	0.07
Gender (female)	OR (CI)	Ref.	1.46 (0.90–2.37)	0.53 (0.24–1.17)	1.20 (0.35–4.07)	1.20 (0.49–2.92)	0.94 (0.54–1.63)
	*p*		0.12	0.11	0.77	0.69	0.82
Residence area (rural)	OR (CI)	Ref.	0.78 (0.50–1.21	0.59 (0.27–1.30)	0.49 (0.15–1.55)	0.35 (0.14–0.84)	0.57 (0.34–0.98)
	*p*		0.26	0.19	0.22	**0.02**	**0.04**
Not married/cohabiting	OR (CI)	Ref.	1.24 (0.75–2.05)	0.47 (0.21–1.03)	1.36 (0.36–5.14)	1.36 (0.51–3.58)	0.59 (0.33–1.03)
	*p*		0.40	0.06	0.65	0.54	0.06
Living alone	OR (CI)	Ref.	3.38 (1.99–5.72)	5.88 (2.57–13.46)	6.01 (1.87–19.27)	7.72 (3.14–19.0)	2.93 (1.58–5.41)
	*p*		** < 0.001**	** < 0.001**	**0.003**	** < 0.001**	**0.001**
Education (secondary or above)	OR (CI)	Ref.	1.98 (1.14–3.46)	3.24 (1.37–7.66)	3.32 (1.01–10.94)	2.36 (0.93–5.99)	2.60 (1.39–4.89)
	*p*		**0.02**	**0.007**	**0.049**	0.07	**0.003**
Economic status (poor)	OR (CI)	Ref.	0.78 (0.46–1.34)	1.31 (0.53–3.24)	1.25 (0.35–4.51)	1.17 (0.46–2.97)	0.75 (0.37–1.49)
	*p*		0.37	0.55	0.73	0.74	0.41
Subjective health (poor)	OR (CI)	Ref.	0.26 (0.16–0.43)	0.64 90.29–1.42)	0.60 (0.19–1.92)	1.82 (0.77–4.32)	0.89 (0.52–1.53)
	*p*		** < 0.001**	0.27	0.39	0.17	0.68
Mobility impairment (homebound)	OR (CI)	Ref.	0.09 (0.04–0.22)	0.24 (0.07–0.82)	0.65 (0.17–2.44)	1.58 (0.68–3.67)	0.54 (0.28–1.02)
	*p*		** < 0.001**	**0.02**	0.52	0.29	**0.06**
Dementia	OR (CI)	Ref.	0.39 (0.24–0.64)	0.67 (0.30–1.50)	0.59 (0.18–1.89)	0.73 (0.30–1.77)	0.54 (0.31–0.97)
	*p*		** < 0.001**	0.33	0.38	0.48	**0.04**
Depression	OR (CI)	Ref.	0.29 (0.18–0.47)	0.42 (0.19–0.93)	0.19 (0.05–0.71)	1.42 (0.58–3.45)	0.45 (0.26–0.78)
	*p*		** < 0.001**	**0.03**	**0.01**	0.45	**0.004**
Functional Disability Index*	*β* (CI)	Ref.	− 0.42 (− 5.07–− 3.14)	− 0.19 (− 5.13–− 1.76)	− 0.13 (− 5.89–− 1.09)	− 0.07(− 3.28–0.51)	− 0.17 (− 3.26, − 0.91)
	*p*		** < 0.001**	** < 0.001**	**0.004**	0.15	**0.001**
Number of chronic diseases*	*β* (CI)	Ref.	− 0.11 (− 0.48–0.39)	0.02 (− 0.62–0.91)	0.01 (− 0.99–1.23)	0.1 (0.001–1.62)	− 0.01 (− 0.61–0.45)
	*p*		0.84	0.71	0.83	0.05	0.78
Number of drugs*	*β* (CI)	Ref.	0.05 (− 0.63–0.74)	0.99 (− 0.22–2.20)	0.13 (− 1.62–1.88)	0.71 (− 0.58–1.99)	0.27 (− 0.56–1.09)
	*p*		0.88	0.11	0.88	0.28	0.53

*In the case of quantitative variables linear regression was used; *β* standardized linear regression coefficient; *OR* odds ratio; *CI* confidence interval; *FD* Family dependent; *LI* Locally integrated; *LS-C* Local self-contained; *WCF* Wider community focused; *PR* Private restricted; *U* Unclassified

Significant differences are marked in bold. In all analyses, a two-tailed *P* value of less than 0.05 was regarded as significant

## Discussion

The frequency of different types of SSNs among community-dwelling people in advanced old age in our study was broadly in keeping with the results of previous research (Wenger 1994a) and confirms the suitability of the PANT as an instrument for assessing support networks of older people in Poland. The most prevalent support networks were “family dependent” (35.8%) and “locally integrated” (32.2%), the former being significantly more common in rural areas. In contrast to our findings, a national, cross-sectional telephone survey in Ireland of people aged 65+ found that the vast majority of respondents (73.2%) were in locally integrated SSNs, rather than family dependent ones, which constituted only 6.9% (Drennan et al. 2008). A similar share of locally integrated networks was observed in population studies among people over 65 in Dublin (Golden et al. 2009). These networks are recognized as among the more robust in terms of their ability to provide appropriate support and facilitate the independence of the older person for longer, by providing the broadest range of options for gaining access to external resources when needed (Hirsch 1980), thus acting as a buffer against the effects of increasing frailty (Wenger 1997a).

The inverse proportions for the share of family-dependent and locally integrated networks between Ireland and Poland may be due to differences in the characteristics of the populations studied, mainly related to age and economic structure and the consequent differences in health status. As observed in earlier studies, the type of support network is correlated with the socio-demographic characteristics of the older person (Wenger 1994a, 1997b), but also, as confirmed in our study, with their health and functional abilities. Higher rates of poverty may also determine the fact that older people live with their children, thus shaping the household structure, which in turn, determines the older person’s network. The greater prevalence of family-dependent networks in our study might reflect higher levels of dependency among the older people studied, together with greater impoverishment, although the latter did not differ significantly among networks in our sample, being almost universally at the lower end of the economic spectrum.

The characteristics significantly more often associated with family-dependent SSNs included more advanced age (80+ years), living with others in rural areas, having poorer levels of education suffering from various disabilities, and a poor self-assessment of health status. Other studies have confirmed that despite a societal shift towards non-kin support networks, it is still the case that family-dependent networks continue to provide informal care among more functionally dependent older adults (Suanet et al. 2019). Indeed, in Poland, there is a cultural imperative for sustaining family care, not least because older people frequently co-reside with their children, and there exists a powerful tradition that daughters in particular, should provide care for their parents (Synak 2002), perceptions shared in other Eastern and Southern European countries (World Bank 2015). These traditions are particularly strong in rural areas (Bien et al. 2007; Wóycicka and Rurarz 2007). Institutionalized care remains an option that is rarely used, only partly because of limited availability. The provision of needs targeted services in the form of residential, and home care is among the lowest in the European Union (European Commission 2015). In 2008, approximately 0.9% of the Polish population over the age of 65 received long-term care in an institutional setting, well below the OECD average of 4.2% (OECD 2011). In 2013, of the dependent population, only 3.4% were receiving formal care in residential institutions (World Bank 2015). A major barrier to good institutional care is that it is almost prohibitively expensive. The Podlaskie region has the lowest provision of formal residential care and the lowest number of older people in residential care in Poland, despite having the highest share of the elderly population in the country. This may partly explain the high prevalence of family-dependent networks in our study.

Locally integrated networks constituted the second most common type of SSN in our study, the health and functional state of older people with this type of network being reliably better than in the case of other SSNs. They were more likely to be living alone and less frequently burdened with disabilities. Our results are consistent with the findings of Thiyagarajan et al. (2014), who confirmed that locally integrated SSNs were less frequently associated with loneliness, depression, self-reported unhappiness, poor self-rated health, disability and care needs.

Other types of support networks, such as “local self-contained”, “private restricted” and “wider community focused” were observed less frequently. Local self-contained SSNs have been found to be more common in areas of low population density, or amongst older people born in such areas (Wenger 1991); the relatively low frequency of these networks is in keeping with the fact that the areas under study were neither remote nor secluded in any way. In particular, the last of these, the wider community-focused network was found with the same frequency as in the study by Golden et al. (2009), but with a lower frequency than observed by Drennan and co-workers (2008).

According to Wenger (1994a), all types of network occur in all communities, though the distribution of network types is related to the socio-demographic characteristics of the community. In our study, both the family-dependent and locally integrated SSNs were significantly more frequently observed in rural areas (73.8% vs 62.4%), whereas wider community-focused and private-restricted SSNs, typical for communities with high population turnover, constituted 11% of support networks in the urban areas and only 5.7% in the rural areas. These differences, however, do not necessarily lie along the rural–urban continuum, but are associated with population stability, the major factor affecting this being migration (Wenger and Leger 1992). Communities with stable populations have a higher proportion of family-dependent and locally integrated SSNs, whereas communities with high population turnover have higher proportions of wider community-focused and private-restricted ones. It is reasonable to suspect that differences in migration history, might at least in part, explain the apparent differences in the distribution of urban vs. rural networks. At the time of our study, the residential mobility of people in this part of Poland was low, and among the older participants of the study, would have been even lower in preceding years (Kaluza 2006). This could explain the low levels of private-restricted and wider community-focused SSNs observed, the more so in rural areas. Networks of this type have been described as “middle class adaptations” (Wenger 1991), and this too may explain why so few occurred in our study, as the socio-demographic background of the participants was almost universally agricultural or working class, as demonstrated by the low educational level of the respondents. Very low levels of mobility would be expected among these societal groups, especially under communism, where people formed closely knit local groups, and have aged in place rather than moving to the area after retirement (Central Statistical Office 2003).

Unclassified SSNs constituted 17.2% of all cases, the most frequently observed borderline type being the family-dependent—locally integrated one, and the family-dependent—private-restricted type. Where borderline cases occur between two network categories, it is likely that the network is shifting from one type to another, in which case, a crisis situation frequently occurs, where the older person's health status worsens, or s/he or key network members change residence, resulting in an altered network structure (Scharf 1997). For older people in Poland, forced migrations resulting from deteriorating health conditions and the need to obtain support and care have dominated patterns of internal migration (Kaluza (2006).

The older person relies on his/her SSN not only to provide day to day help with personal care and to facilitate engagement in everyday life, but also for emotional support, companionship and advice. The extent to which this support is available depends on the combination of relationships in any particular network. The more remote the connection, the more symbolic the expectations; the closer the connections, the wider the scope for normative expectations. This is also influenced by the quality of former mutual ties and reciprocity (Bruggencate et al. 2018), as well as the quality of help provided, geographic proximity, age, gender and health status of network members (Ashida and Heaney 2008). Different types of networks have been shown to have different strengths and weaknesses, and the nature of potential risks, such as loss of independence or breakdown of home care, is related to the type of SSN. For example, although family-dependent networks, most prevalent in the present study, have obvious benefits for the older person, maintaining kinship ties and providing care even at high dependency levels, they are often small in size and inevitably limited in expertise. They are thus associated with a narrower range of resources than in networks with a more heterogeneous membership (Hammer 1983). Hence, they risk being unable to provide the older person with the range of interventions required, especially where supplemental assistance from formal home care services is limited, as is the case in Poland (World Bank 2015). Furthermore, it has been shown that loneliness is more common in networks which are primarily made up of kin (Dykstra 1990). It is particularly important that the carers in family-dependent support networks, receive appropriate support to help them function efficiently and in good health against the toll that the provision of constant care to their older family members requires (Bien 2006; Wojszel 2009).

Specific problems such as dementia, serious disability or death of a primary caregiver can cause the support network structure to change (Wenger 1994b). The ability of networks to adapt to these changes is important for continued support. The relatively large proportion of networks that could not be classified among the samples studied raises the question of whether these were networks that were in the process of reformulating themselves. They may have been in transition from one of the two most common networks, as most prominent among them were the family-dependent—locally integrated types, suggesting that they might have been undergoing transformation due to increasing disability of the older person or as a result of the inability of the primary caregiver to continue in this capacity. Whatever the case, it is potentially a signal that the network of the older person lacks stability and may be approaching a major change or crisis. Indeed, people with unclassified networks were more likely to be living alone in rural areas and to have severe functional disabilities, often homebound and suffering from depression and dementia, all of which inevitably signal greater risk. These situations are often not recognized because there is no preventive support available and needs assessment remains limited, until just such a crisis develops.

Knowledge of this kind is important for clinical and social care practitioners, as it is relevant to decision-making and interventions, particularly with regard to social isolation, deteriorating health, hospital discharge, admission to residential care and the mental state of the older person. An improved understanding of the prevalence of different types of social networks among older people in Poland would help guide a systematic approach to recognizing unmet needs in this population. However, this might be difficult to enact in practice, as social services are heavily burdened with many administrative activities and frequently able to react only in crisis situations. A more proactive approach is necessary to provide the crucial information required in the planning of formal services.

The structure of the support networks of older people in a given population provide useful information concerning the contribution of informal caregivers and the demand for formal care rendered by social services (Scharf 1997). This offers a practical contribution to the local planning of health and social services for older people. In particular, private-restricted and local self-contained networks are indicative that special attention should be addressed to the older person’s situation, and it may be critical to do so when undertaking decisions or interventions under the circumstances mentioned above (Drennan et al. 2008; Wenger and Tucker 2002). An important factor to bear in mind is that it is not the magnitude of the particular support group that is key to providing support for the older person, but rather the type of connection with the older person supported by the group (Cornwell et al. 2008; Li et al. 2014), as well as the culturally associated obligations and expectations for different types of members of the network (Burholt and Dobbs 2014; Rodrigues et al. 2014). In Poland, the cultural expectation for family care remains strong, both on the part of the older person and his/her children, with the widely held assumption that it should be provided by daughters or daughters-in-law (World Bank 2015).

Summing up, the results of the study support other authors’ findings that the network type is associated with the socio-demographic characteristics of older people and with health outcomes (i.e., subjective well-being, cognitive ability, depression, disability level) (Thiyagarajan et al. 2014; Wenger 1994a). To the authors’ knowledge, this is the first publication in the literature using the PANT SSN typology in 75+ year-old people in Poland.

There are nonetheless certain limitations to this study, resulting from the research methods used. Random quota sampling may have influenced the representativeness of the findings, despite the sample being matched exactly in terms of age and gender to the demographic structure of the population in the areas studied (Central Statistical Office 2008), and similar distributions for other variables having been previously reported (Synak 2002; Wojszel 2009). Furthermore, certain data concerning the prevalence of chronic diseases and functional ability were based on self/carer reports, which may lack accuracy. It has been demonstrated that there are sometimes differences between declared and observed functional abilities for particular tasks (Kempen et al. 1996). Moreover, assessment of cognitive function and emotional status was based on screening tests only, and therefore did not represent a definite diagnosis. Finally, the fact that the research was cross-sectional, providing a statistical description of the situation of older people, means that it is unable to explain cause-and-effect relationships nor take into account the variations that can occur in support networks over time. For this reason, it would be valuable to re-evaluate the questions raised in this study from a longitudinal perspective.

## Conclusions

The results of the study support other authors’ findings, confirming that the network type is associated with the socio-demographic characteristics of older people, and with their health outcomes. The two most frequently observed SSN types in people of advanced old age in the areas studied in Poland were family-dependent and locally integrated ones. Of these, the latter is recognized in the literature as one of the most robust types, correlating differentially with the functional disability of the respondents and enabling people to maintain their independence for longer. By contrast, the family-dependent network had significantly higher odds for functional and cognitive disability, depression and living in rural areas than other types of support network.
